# The Effects of Workplace-Based HIV Self-testing on Uptake of Testing and Linkage to HIV Care or Prevention by Men in Uganda (WISe-Men): Protocol for a Cluster Randomized Trial

**DOI:** 10.2196/25099

**Published:** 2021-11-01

**Authors:** Patience A Muwanguzi, Tom Denis Ngabirano, Noah Kiwanuka, LaRon E Nelson, Esther M Nasuuna, Charles Peter Osingada, Racheal Nabunya, Damalie Nakanjako, Nelson K Sewankambo

**Affiliations:** 1 School of Health Sciences College of Health Sciences Makerere University Kampala Uganda; 2 School of Public Health College of Health Sciences Makerere University Kampala Uganda; 3 Yale School of Nursing Yale University New Haven, CT United States; 4 Infectious Diseases Institute College of Health Sciences Makerere University Kampala Uganda; 5 Department of Infectious Diseases Research African Center for Health Equity Research and Innovation Kampala Uganda; 6 School of Medicine College of Health Sciences Makerere University Kampala Uganda

**Keywords:** Africa, workplace HIV testing, HIV self-testing, linkage to care, linkage to prevention

## Abstract

**Background:**

HIV testing uptake remains low among men in sub-Saharan Africa. HIV self-testing (HIVST) at the workplace is a novel approach to increase the availability of, and access to, testing among men. However, both access and linkage to posttest services remain a challenge.

**Objective:**

The aim of this protocol is to describe a cluster randomized trial (CRT)—Workplace-Based HIV Self-testing Among Men (WISe-Men)—to evaluate the effect of HIVST in workplace settings on the uptake of HIV testing services (HTS) and linkage to treatment and prevention services among men employed in private security services in Uganda.

**Methods:**

This is a two-arm CRT involving men employed in private security services in two Ugandan districts. The participants in the intervention clusters will undergo workplace-based HIVST using OraQuick test kits. Those in the control clusters will receive routine HTS at their work premises. In addition to HTS, participants in both the intervention and control arms will undergo other tests and assessments, which include blood pressure assessment, blood glucose and BMI measurement, and rapid diagnostic testing for syphilis. The primary outcome is the uptake of HIV testing. The secondary outcomes include HIV status reporting, linkage into HIV care and confirmatory testing following HIVST, initiation of antiretroviral therapy following a confirmatory HIV test, the uptake of voluntary medical male circumcision, consistent condom use, and the uptake of pre-exposure prophylaxis by the most at-risk populations.

**Results:**

Participant enrollment commenced in February 2020, and the trial is still recruiting study participants. Follow-up for currently enrolled participants is ongoing. Data collection and analysis is expected to be completed in December 2021.

**Conclusions:**

The WISe-Men trial will provide information regarding whether self-testing at worksites increases the uptake of HIV testing as well as the linkage to care and prevention services at male-dominated workplaces in Uganda. Additionally, the findings will help us propose strategies for improving men’s engagement in HTS and ways to improve linkage to further care following a reactive or nonreactive HIVST result.

**Trial Registration:**

ClinicalTrials.gov NCT04164433; https://clinicaltrials.gov/ct2/show/NCT04164433

**International Registered Report Identifier (IRRID):**

DERR1-10.2196/25099

## Introduction

### Background

Global estimates report that 81% of people living with HIV (PLHIV) knew their HIV status at the end of 2019, and 67% were on antiretroviral therapy (ART) [1]. Over the past several years, there has been a significant decrease in new HIV infections and an increase in the proportion of people accessing ART, with a consequent decline in AIDS-related deaths [2]. The decline in HIV/AIDS-related deaths is attributable, at least in part, to early initiation of HIV care and improved adherence to ART [2]. While numerous efforts and advances in the fight to end the HIV/AIDS epidemic have resulted in substantial gains, the HIV prevalence of 6.3% in Uganda is still quite high [3]. In 2019, there were approximately 1.4 million PLHIV, and approximately 23,000 died of AIDS-related illnesses in Uganda [4].

The failure to reach greater numbers of men with HIV testing and treatment appears to be driving ongoing cycles of HIV transmission in different settings [5]. Not surprisingly, in Uganda, more women (86%) than men (78%) know their HIV status [6]. This may be partly because, unlike women who attend regular maternity and reproductive health services, men do not have similar touch points within the health care system [7]. Furthermore, there are overlooked gender norms and societal beliefs around masculinity and health testing behaviors [8,9] as well as increasing homophobia and transphobia at health facilities [10,11]. When men living with HIV are not diagnosed in a timely fashion, do not start treatment, or fail to remain on treatment, it endangers not only their own health but also the well-being of their families and communities [12]. The current level of new infections in Uganda is still remarkably high, with an estimated 53,000 newly infected people in 2019 [4]. This may be an indication that the country will continue to register high numbers of people with HIV unless innovative measures are put in place to reach and test hard-to-reach populations such as men and youths [13]. Recognizing the importance of closing this gap, various efforts are being directed at developing innovative strategies to increase men’s engagement in HIV prevention and care.

The Joint United Nations Programme on HIV/AIDS (UNAIDS) campaign “Blind spot: Reaching out to men and boys” encourages HIV programs to design campaigns that promote men’s engagement in HIV services [12]. In line with HIV testing, men in different parts of Uganda have expressed concern with getting tested at a health facility because of the long queues, poor attitudes of health workers, fear of being labeled an HIV “victim,” and stigma [14,15]. Additionally, some men have declined an HIV test for fear of imminent death following a positive result, fear that a positive result would stop them from acquiring new sexual partners, fear of a divorce, and a lack of confidentiality for the test results [16-18]. In that regard, the Uganda National HIV Testing Services Policy and Implementation Guidelines [19] proposed that men be targeted at their workplaces, because that is where they spend most of their time. The goal of workplace HIV testing services (HTS) is to increase access to HIV testing by men and women. This approach has demonstrated success in increasing the uptake of HTS at several male-dominated worksites [6].

We propose the use of HIV self-testing (HIVST) to increase the uptake of HTS at the workplace. HIVST overcomes some of the barriers for standard HTS, such as stigma, long lines at health facilities, lack of time to take a test, and the perceived lack of confidentiality of test results [20,21]. During HIVST, an individual collects his or her own oral fluids or blood, performs the test either in private or with someone he or she trusts, and then interprets the results [22]. According to the World Health Organization (WHO), several studies have demonstrated the feasibility and acceptability of HIVST in diverse populations, including men [23]. In a study conducted in South Africa, Van Dyk observed that participants who preferred HIVST were predominantly men [24]. In Malawi, Zambia, and Zimbabwe, Hatzold and colleagues used several HIVST distribution models among men, young people, and index HIV testers [25]. Current trends indicate that HIVST is gaining traction, and many countries have developed policies and guidelines for large-scale implementation [26]. Therefore, this study aims to assess the effectiveness of HIVST in increasing the uptake of HIV testing among wen in work settings in Uganda. However, even as evidence of the high feasibility, acceptability, and accuracy of HIVST continues to accumulate across many delivery models, there is a need for randomized trials to evaluate the outcomes and cost-effectiveness of different HIVST delivery models. More importantly, trials that focus on the linkage to HIV prevention and treatment remain a necessity.

### Objective

We present the protocol for the Workplace-Based HIV Self-testing Among Men (WISe-Men) study, which is a cluster randomized trial (CRT) to assess the effectiveness of workplace-based HIVST in Uganda. The aim of this trial is to evaluate the effect of HIVST in work settings on the uptake of HTS and linkage to further HIV services among men employed in private security services in Uganda.

### Hypothesis

We hypothesize that workplace-based HIVST will increase the proportion of men who take an HIV test, the proportion of men who initiate ART following a positive result, and the proportion of men who are linked to prevention services following a negative result.

## Methods

### Conceptual Model

**Figure 1:**
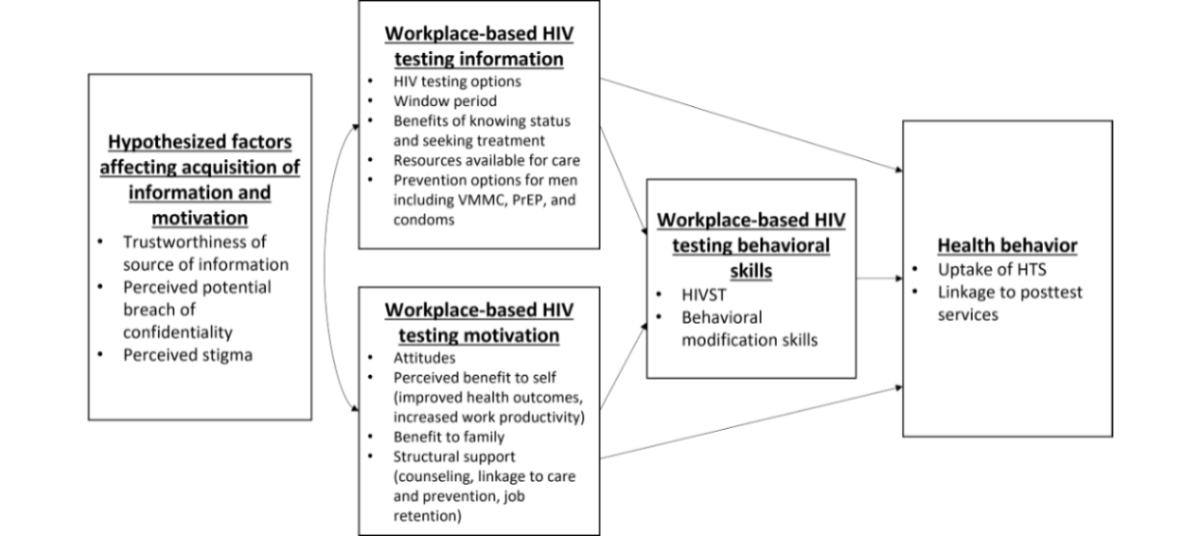
Conceptual model adapted from the information-motivation-behavioral skills model. HIVST: HIV self-testing; HTS: HIV testing services; PrEP: pre-exposure prophylaxis; VMMC: voluntary medical male circumcision.

This trial is guided by the information-motivation-behavioral skills (IMB) model [27]. The model suggests that health-related sensitization, incentives, and behavioral skills are key determinants for health behaviors. It asserts that if individuals are well informed, receive the appropriate motivation, and have the right behavioral skills, they are more likely to initiate and sustain behaviors that lead to positive health outcomes [28,29]. We use a modified version of the IMB model proposed by a study for HIV testing in the emergency department [28,30] (Figure 1).

### Study Setting

This study will be conducted in two Ugandan districts, namely Kampala, the capital city, which houses headquarters for most companies offering security services in Uganda, and Hoima, an oil and natural gas base that has several security companies. Additionally, the social and relational dynamics created by the work demands of men working in private security services have an influence on their vulnerability to HIV risk. Many of the workers migrate from their homes to work in cities, which places them at high risk of HIV, especially if they remain away from home and/or away from their regular partners for long periods [31].

### Study Participants

Permission will be sought from the management of each company to allow the research team to meet with the employees at the company premises. The team will meet the employees during the morning meeting to introduce the study and provide information leaflets. The team will then return on another agreed-upon day to enroll participants, collect baseline data, and carry out the workplace HIV testing. On each data collection day, all eligible employees will receive an equal opportunity to participate in the study activities. Initially, the men will receive study information as a group, followed by one-on-one eligibility assessment and subsequent recruitment.

### Study Design

This is a two-arm CRT involving men employed in private security companies. Clusters were private security companies employing 50 or more men in two districts in Uganda. This CRT was informed partly by findings from a previous exploratory qualitative study exploring the perceptions and preferences of employers and employees in private security companies regarding workplace-based HIVST and linkage to further services [32]. The study proposed several strategies to optimize linkage to posttest services following workplace-based HIV testing. These strategies included the use of referral and linkage documentation, paid time off from employers to attend health facilities, assurance of the confidentiality of the test results, peer support from PLHIV, health education and sensitization, expanded clinic hours, the reduction of stigma, and the mitigation of any potential harm. Furthermore, both the employers and employees in the security companies proposed the inclusion of further assessments in addition to HIV testing. This approach would allow them to understand their health status and reduce the stigma associated with taking an HIV test more fully. The additional health assessments include measuring blood pressure and blood glucose, assessing BMI, and screening for syphilis. Participation will be discontinued in response to harm or participant request.

### Site Selection and Allocation

Through randomization, Kampala District was allocated to the intervention arm and Hoima District was allocated to the control arm. The clusters in the intervention arm will receive HIVST while those in the control arm will receive standard HTS.

### Eligibility Criteria

Eligible private security companies, each employing at least 50 male personnel, were identified and listed per district. The eligibility criteria for participants are as follows [33]:

- Men 18 to 60 years old
- Employed more than 6 months within the security industry
- Men who have either never taken an HIV test or who tested negative for HIV more than 1 year ago.

### Ethics Approval, Trial Registration, and Informed Consent

Both the Makerere University School of Health Sciences Research Ethics Committee (reference No. 2018-054) and the Uganda National Council of Science and Technology (UNCST; reference No. HS 2672) granted ethics approval. The trial was registered at ClinicalTrials.gov (NCT04164433). Any important protocol deviations or adverse events (AEs) will immediately be communicated to the Research Ethics Committee, the UNCST, and ClinicalTrials.gov. Additionally, we will seek permission from the responsible personnel officer at every site.

Each participant will provide written consent prior to recruitment into the CRT and will receive a copy of the signed form. They will also be informed that their participation is voluntary and that they may withdraw from the study at any time. Furthermore, permission will be sought to audio-record and take notes during the interviews.

All disclosed HIV status results will remain confidential. The employers will be made aware that the results will remain confidential and that the workers are not under any obligation to disclose their results, especially in the event that this may lead to a loss of jobs for those who are found to be HIV positive. All original documents will be deposited in a secure locked cabinet and accessed only by the three investigators. The trial data set will not have any participant identifiers and will be stored in electronic password-protected files.

### Sample Size Determination

Sample size estimation is based on the primary outcome: proportion of men who take an HIV test. The main outcomes were at the participant level. Currently, approximately 55% of males in Uganda have taken an HIV test [34]. We hypothesize a 15% increase in HIV testing in the workplace HIVST group in comparison to the control group. We considered a two-sided α of .05 with a power of 80% to detect a significant change between both groups, a 1:1 allocation ratio between the intervention and control arms, a response rate of 90%, and a design effect of 1.399. We estimate a minimum total sample size of 548 participants, with 274 per arm. The proxy for design effect was picked from the proxy variable “Had taken an HIV test and obtained results in the past year” [35]. 

### Data Collection

#### Cluster Randomized Trial Flow

**Figure 2:**
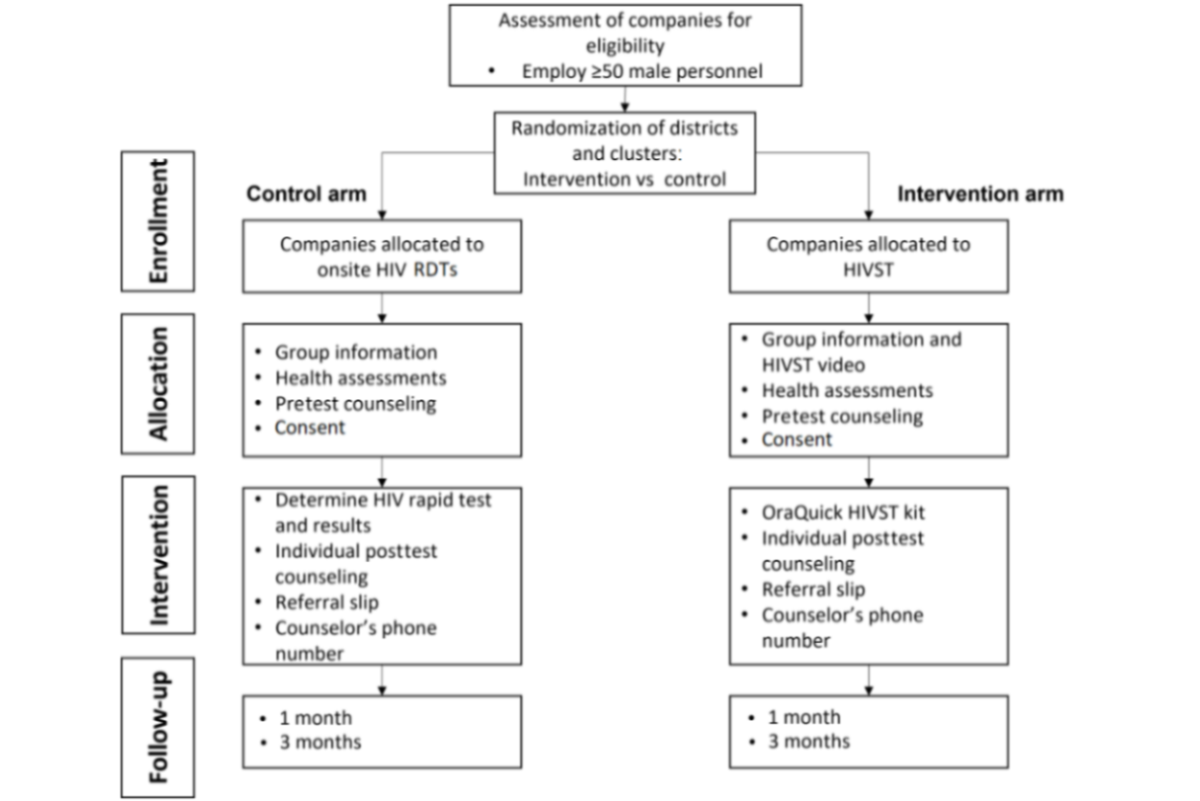
Cluster randomized trial flow diagram. HIVST: HIV self-testing; RDT: rapid diagnostic test.

All participants will be provided with a list of nearby health facilities that are accredited to provide ART. Participants will be asked to propose three facilities that they would like to visit for a confirmatory test if found to be HIV positive (Figure 2).

#### Intervention Arm

**Figure 3:**
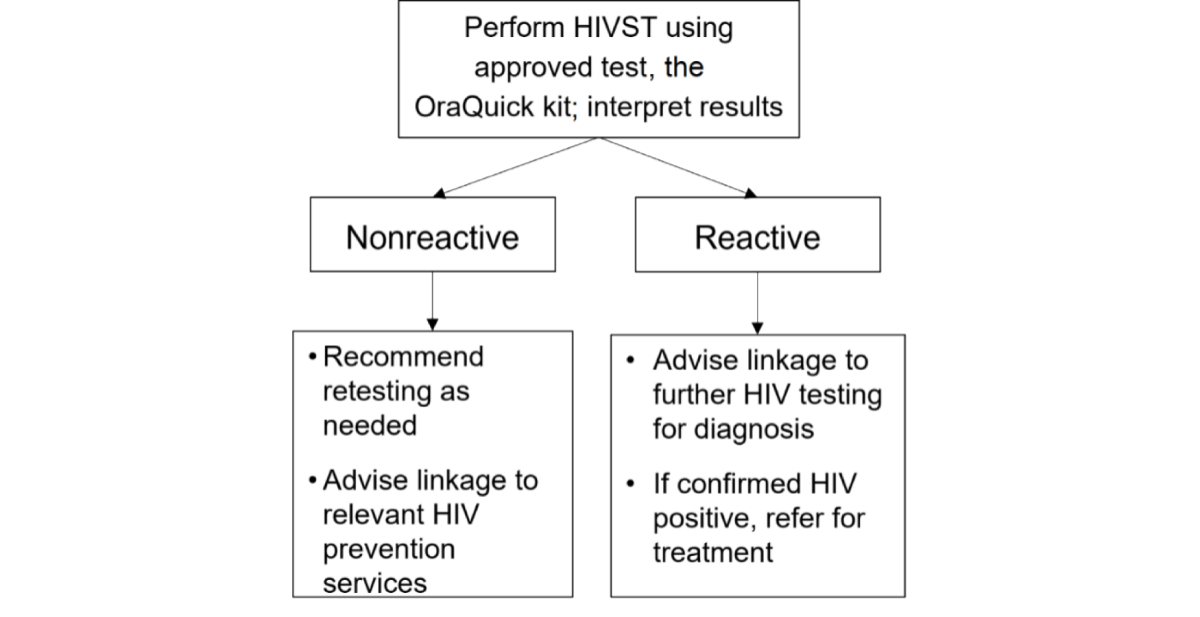
HIV self-testing (HIVST) pathway.

Since 2014, the WHO has encouraged countries to implement pilot HIVST programs to evaluate this approach. We will, therefore, follow the WHO strategy on self-testing to pilot HIVST in the workplace [22] (Figure 3). The strategy steps are as follows:

1. Explain the HIVST procedure and how to interpret the self-test results.
2. Demonstrate the HIVST procedure and the interpretation of the results.
3. Provide any additional instructional materials using an HIVST video.
4. Answer any questions raised by the potential participant.
5. Issue the OraQuick HIVST kits for collection of a mouth swab; participants will test at home or at a venue of their choice.
6. Participants will return the results to the trial team in one of the following ways that will be agreed upon when they receive the kit:
  - A phone call to the study toll-free line.
  - A picture of the test results sent via email, WhatsApp, Facebook, or another preferred social media app.
  - Presentation of the test kit at the health facility.
  - Participant self-reporting the results at the health facility.
7. Provide a referral slip and further information on linking to further services for those who receive a reactive self-test result. Those who test negative will receive further information and a referral to HIV prevention services.
8. Provide participants with a counselor’s phone number. They will be offered the option of a call-back to return the results within 3 days of testing. Participants will also be told to use this number if they have any difficulty and need support during and after testing. This is in line with a recommendation from a study in Nigeria [36], where more than half of the participants who tested HIV positive used the helpline for support.
9. Each participant will receive a code and instructions on what to write in case he is positive and can send these results as a text message. For example, 2018-7TR9-122-S is the code for a positive reactive test, and 2018-7TR9-122-T is the code for the nonreactive test. These codes will be randomly generated for each participant to avoid coworkers being able to accidentally interpret another person’s results.
10. Participants who do not return the HIVST results within 3 days shall be phoned. This is in line with the Ugandan Ministry of Health test-and-treat strategy [6]. They will have given prior consent to be contacted.

#### Control Arm

**Figure 4:**
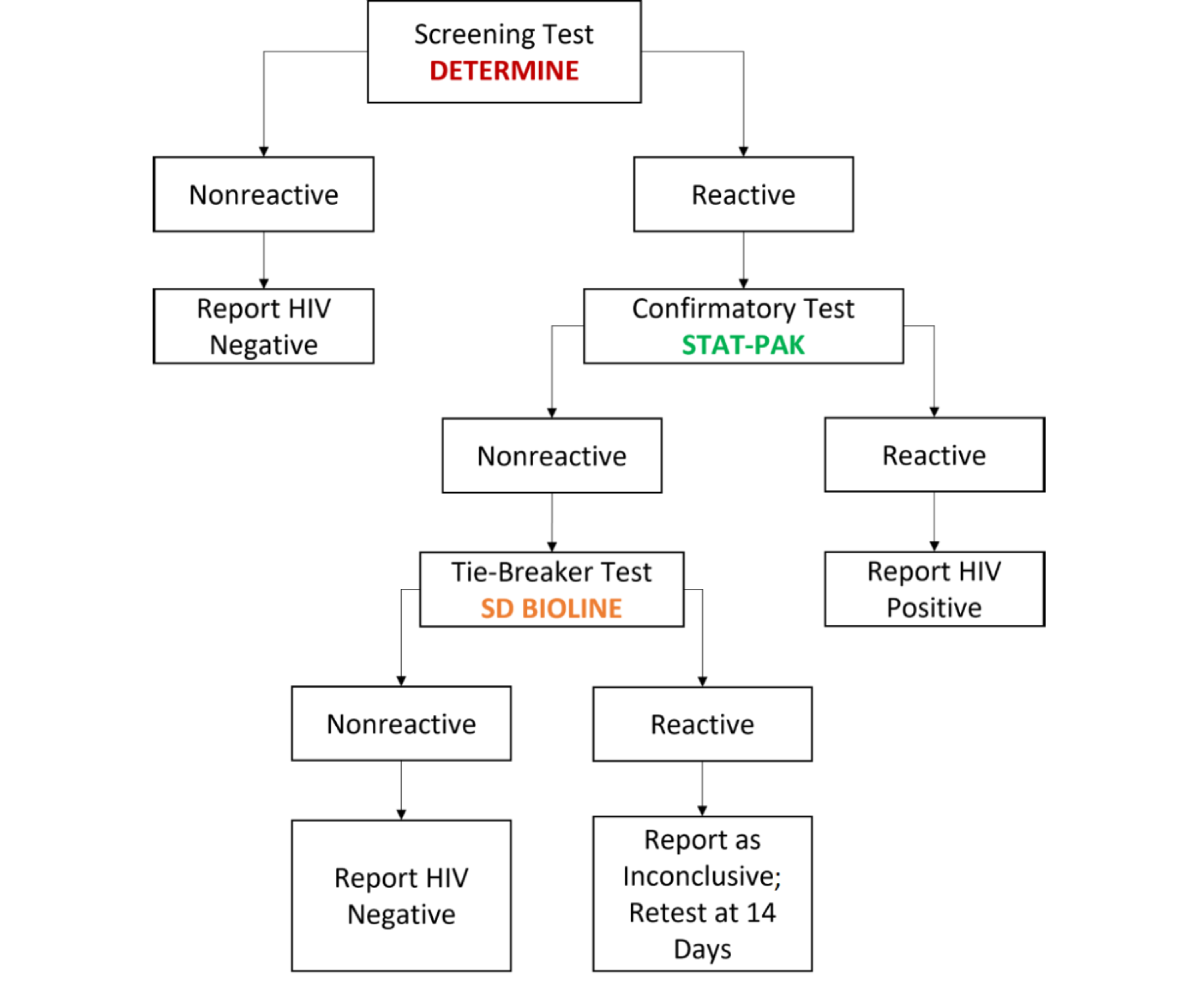
Serial HIV testing algorithm for testing persons over 18 months of age in Uganda.

Security personnel allocated to the control arm will receive standard-of-care HIV counseling and testing based on the Ugandan Ministry of Health guidelines [6], as follows:

1. The control arm of this study will be offered standard facility-based care using antibody-based rapid diagnostic tests (RDTs), which follow the approved serial testing algorithm in Uganda for people above 18 months of age [6] (Figure 4).
2. Whole blood will be collected via finger stick and capillary sampling by a trained HIV nurse or counselor who will use a lancet and transfer the blood to the screening test kit using a capillary tube. The specimen will be tested immediately.
3. The nationally approved algorithm for HIV testing is as follows:
  - All participants will undergo the screening test using the Alere Determine HIV-1/2 test (Abbott).
  - All participants who receive a reactive result will undertake a confirmatory test using the HIV 1/2 STAT-PAK test (Chembio Diagnostic Systems).
  - All participants with a nonreactive confirmatory test result will undertake a tie-breaker test using the SD BIOLINE HIV-1/2 test (Abbott).
4. Participants whose samples react using the tie-breaker test shall be retested after 14 days.
5. Participants who test positive will receive a referral slip to nearby public and private health facilities to expedite linkage to treatment, care, and support services.
6. Participants who test negative will receive a referral slip for HIV prevention services. The participants will be referred to one of five public health facilities within the district.
7. Each participant will be provided with a counselor’s contact number for continued consultation.

### Linkage to Care or Prevention Services

The participants will be followed up at 1 month and 3 months to determine their linkage into prevention, treatment, or care.

#### Referral of Clients With a Confirmed HIV Diagnosis

Following confirmatory HIV testing, clients diagnosed with HIV will be referred to local HIV treatment facilities using a paper referral slip provided by the research team. Once a client presents to a health facility with a referral slip, they will receive a confirmatory test prior to ART initiation. This approach reduces the risk of misdiagnosing their HIV status and unnecessarily treating HIV-negative persons, which has ethical and health system implications and causes individuals to suffer needless psychological effects [6,37]. A copy of the referral slip will be retained by the research team for use during follow-up. Participants who test positive for HIV will be encouraged to take their referral slip to the referral facility where staff will have been trained to keep the referral documentation and mark the source of the referral during patient registration. The unit of evaluation (ie, the indicator for the outcome of linkage to care) will be defined by the facility’s HIV testing registration records and clinic records of ART initiation.

#### Referral of Clients Confirmed as HIV Negative

Following confirmation of an HIV-negative result, clients will be offered information about voluntary medical male circumcision (VMMC). Pre-exposure prophylaxis (PrEP), condom use, and partner notification will be offered as an option for those who are sexually active. They will also receive a referral slip for these services to refer them to the nearest public health facility of their choice from their company offices. As mentioned above, the clients who present at the health facility will also receive a confirmatory test. The unit of evaluation will be defined by HIV testing by the facility, VMMC, partner services, and PrEP records.

#### Permitted Concomitant Interventions

In addition to HTS, security personnel in both the intervention and control arms will receive identical additional services. These services were identified through a needs assessment that was conducted earlier [32]. The findings from that study reported that both employers and employees were more willing to undergo HIV testing at the workplace if this was provided in combination with other health promotion interventions as a way of mitigating stigma. The additional package will, therefore, include the following: a blood pressure assessment, blood glucose and BMI measurements, and an RDT for syphilis. The additional package will be offered to both the intervention and control groups prior to enrollment in the study. We do not expect these concomitant interventions to affect the results of the trial.

### Study Outcomes

#### Overview

The primary outcome will be uptake of HIV testing. Secondary outcomes will include testing yield, proportion of participants initiating ART during the first 3 months following HIV test results, and proportion of participants linked to prevention services (ie, consistent condom use, VMMC, PrEP, and retesting for HIV).

#### Primary Outcome Assessment

We are operationally defining uptake of HIV testing as accepting to take an HIV test from the standard-of-care services that will be offered to the control group or receiving and returning a used HIVST kit for the intervention group. The proportion of security personnel in the control group who take an HIV test will be computed by dividing the number of participants in the standard-of-care cluster who agree to take an HIV test by the total number of those enrolled in the control arm. For the participants in the intervention cluster, uptake of HIV testing will be computed by dividing the number of participants who return the used HIVST kit within 1 month from the time of the test kit distribution by the total number who enrolled in the intervention arm.

#### Secondary Outcome Assessment

The following outcomes will be reported in the clinical trial record [33]:

1. HIV status reporting—the proportion of participants who self-report HIV test results. This will be assessed via telephone call by participants to the trial toll-free line or via picture of the self-test result sent through any of the following electronic channels: study email, WhatsApp, or the study Facebook account. Further assessment will include the presentation of either the trial participant or the self-test at a health facility.
2. Linkage into HIV care—the proportion of participants with positive results who link to a health facility for HIV care. This will be assessed by checking the clinic records from the selected HIV care and treatment facilities at 1 and 3 months.
3. Initiation of ART—the proportion of participants who initiate ART. This will be assessed by checking clinic records or possession of an ART card. Data will be collected using a questionnaire at 1 and 3 months.
4. Uptake of VMMC following HIVST—the proportion of previously uncircumcised participants who self-report VMMC at 1 and 3 months.
5. Consistent condom use following HIVST—the proportion of participants who use a condom for each sexual encounter for 1 month. Condom use will be assessed through verbal reports from the participants. Participants will be followed up at 1 and 3 months.
6. Uptake of PrEP by men who have sex with men (MSM)—the proportion of MSM who initiate PrEP at 1 and 3 months. In this study, MSM will indicate the behaviors that transmit HIV infection, rather than how individuals self-identify in terms of their sexuality [38].

### Data Monitoring, Potential Harms, and Audit Process

HIV testing and HIVST at the workplace in Uganda are still novel and may result in unexpected harms and AEs. We set up a data safety and monitoring committee to oversee the progress of the trial. The committee is charged with ensuring that the trial protocol is adhered to and that the trial data are correctly recorded, analyzed, and reported. The trial quality-management officer, together with the research team, will monitor the participants for harm and AEs, such as physical violence, stigma, and discrimination at the workplace, as well as personal and social harm related to receiving HIV test results. AEs will be classified as mild, moderate, or severe. AEs such as suicide threats, self-harm threats, hospitalization, or death up to 28 days after a reactive HIVST result will be classified as severe [39]. The trial quality-management officer will report all events and withdrawals from the trial due to AEs using an open-ended AE reporting form. These data will be collected fortnightly. Severe events will be reported to the principal investigator and the Research Ethics Committee, while mild and moderate AEs will be recorded and reported to the trial counseling team. In the event of severe AEs, the data monitoring committee will evaluate the benefits and harms separately, followed by an overall measure that considers the balance between benefits and harms [40]. At the end of the trial, two independent officials will evaluate the trial-related activities and documents to ensure that they were carried out according to the protocol and Good Clinical Practices.

### Statistical Analysis

We will use the intention-to-treat principle for all analyses. Participants’ sociodemographic characteristics will be summarized using frequencies and proportions and will be compared across the study arms. We will employ chi-square and Fisher exact tests for categorical variables as well as means and *t* tests or analyses of variance for continuous variables [41]. To avoid unit-of-analysis errors, we shall conduct analyses by allocation unit via hierarchical logistic regression models that compare the outcome between the two arms and account for clustering among the respondents. The analysis will also adjust for individual-level covariates to elicit associations between the outcomes and the covariates. This method of analysis has been selected over other methods of analyzing cluster data to avoid any ecological fallacy [42]. For the primary outcome, we will compare the proportion of participants taking an HIV test between the two arms. Secondary analyses will be conducted to compare the proportion of HIV-positive participants who become linked to care between the two arms as well as the proportion of HIV-negative participants who take up prevention services between the two arms. The statistical analysis will be performed using Stata software (version 14; StataCorp LP). Trial findings will be published following the CONSORT (Consolidated Standards of Reporting Trials) guidelines [43].

## Results

Participant enrollment for the WISe-Men trial commenced in February 2020 and was still recruiting study participants at the time of this submission. Follow-up for currently enrolled participants is ongoing. Data collection and analysis is expected to be completed in December 2021.

## Discussion

This research project aims to evaluate the effects of HIVST on HIV care-seeking among men. One anticipated challenge of the HIVST intervention is how the researchers will confirm the participants’ HIVST results, as these will be self-reported. We propose the use of mobile phone apps, such as WhatsApp, that participants can use to send their results back for verification, but this may be a challenge for those without smartphones. Another anticipated challenge revolves around participants’ work schedules. The field employees in the private security companies may not be available for HTS on the days scheduled for data collection, which may delay recruitment and hinder achieving the desired sample size.

With 88% of PLHIV identified in Uganda, it is difficult to identify the remaining undiagnosed PLHIV with general population approaches. This project uses one of the recommended approaches called targeted testing, which focuses on an individual or group of individuals who are at high risk of HIV acquisition [6]. Men employed in private security companies represent an ideal population for targeted testing. They are considered among the priority populations with limited access to HTS due to the nature of their work, and their social migration puts them at risk of HIV [34]. Additionally, the WISe-Men trial will provide information regarding whether testing at worksites increases men’s engagement in HIV testing and linkage to post-HTS in Uganda. The trial results will be communicated to the participants, health care professionals, and the public through workshops and meetings and to other relevant groups through publications in peer-reviewed journals and conferences. The findings from this study will contribute to policy development regarding HIVST and will inform the future regional, national, and international implementation of HTS.

## Abbreviations

AE: adverse event
ART: antiretroviral therapy‎
CONSORT: Consolidated Standards of Reporting Trials
CRT: cluster randomized trial
HIVST: HIV self-testing
HTS: HIV testing services
IMB: information-motivation-behavioral skills
MSM: men who have sex with men
PLHIV: people living with HIV
PrEP: pre-exposure prophylaxis
RDT: rapid diagnostic test
UNAIDS: Joint United Nations Programme on HIV/AIDS
UNCST: Uganda National Council of Science and Technology
VMMC: voluntary medical male circumcision
WHO: World Health Organization
WISe-Men: Workplace-Based HIV Self-testing Among Men
